# ATP6V0d2 mediates leucine-induced mTORC1 activation and polarization of macrophages

**DOI:** 10.1007/s13238-019-0636-x

**Published:** 2019-05-27

**Authors:** Pingfei Li, Xiaofei Deng, Jing Luo, Yufei Chen, Guoyu Bi, Feili Gong, Zhengping Wei, Na Liu, Huabin Li, Arian Laurence, Xiang-Ping Yang

**Affiliations:** 1 Department of Immunology, School of Basic Medicine, Tongji Medical College, Huazhong University of Science and Technology, Wuhan, 430030 China; 2 Department of Immunology, School of Basic Medicine, Hubei University of Medicine, Shiyan, 442000 China; 3 The First Affiliated Hospital, Biomedical Translational Research Institute and Guangdong Province Key Laboratory of Molecular Immunology and Antibody Engineering, Jinan University, Guangzhou, 510632 China; 4 Department of Otolaryngology-Head and Neck Surgery, Center for Allergic and Inflammatory Diseases, Affiliated Eye and ENT Hospital, Fudan University, Shanghai, 200031 China; 5 Department of Haematology, University College London Hospitals NHS trust, London, UK

**Dear Editor,**

mTORC1, as a center regulatory hub of metabolism, senses the cellular energy status, nutrition and extracellular stimuli and regulates cell growth, differentiation and functions of immune cells (Powell et al., 2012). Lysosomal localization of key signal components is critical for mTORC1 activation: mTORC1 activation requires co-localization of activated Rheb and mTORC1 to the lysosome membrane (Buerger et al., 2006). Signals including growth factors, cellular stresses and energy levels act on the disruption the formation of tuberous sclerosis complex (TSC) complex, comprised of TSC1, TSC2 and TBC1D7, which leads to the translocation and activation of Rheb on the lysosome membrane (Dibble et al., 2012). In response to nutrient levels, specifically the availability of amino acids and glucose (Efeyan et al., 2013), mTORC1 is recruited to the lysosomal surface by Rag GTPases that are heterodimers of RagA or RagC bound to RagB or RagD. Multiple protein complexes have been implicated in regulation of mTORC1 upon nutrient sensing including Ragulator, GATOR1, GATOR2, KICSTOR and vacuolar ATPases (Wolfson et al., 2017). Vacuolar ATPases are large multiple-protein complexes that acidify the lysosome and may mediate additional functions independent of their proton pump activity (Nishi and Forgac, 2002).

Although a number of amino acid sensors have been identified (Chantranupong et al., 2016), the regulation of mTORC1 activation by amino acids remains largely elusive. Furthermore, the majority of studies examining amino acid-induced mTORC1 signaling were performed in cell lines and little is known about the amino acid- and cell type-specificity of mTORC1 activation.

We recently identified ATP6V0d2 as a macrophage-specific subunit of vacuolar ATPase, whose expression is restricted in macrophages and inhibited by inflammatory stimuli and tumor cell-derived lactate (Liu et al., 2019). ATP6V0d2 inhibits inflammation and bacterial infection by promoting autophagosome and lysosome fusion (Xia et al., 2019). In addition, ATP6V0d2 can mediate HIF-2α degradation, limiting macrophage protumoral activity (Liu et al., 2019). Given the restricted expression of ATP6V0d2 and its localization in lysosome membrane, we speculated that ATP6V0d2 might play a role in amino acid-mediated mTORC1 activation in macrophages.

First, we stimulated amino acid-starved HEK293T cells with increasing amounts of leucine, arginine or glutamine and measured mTORC1 activation. All three amino acids induced mTORC1 activation in a dose-dependent manner, measured by the phosphorylation of ribosome protein S6 and 4-EBP1 (Fig. S1A–C). Next, we used the optimized amino acid concentration (4 mmol/L leucine, 20 mmol/L glutamine, 2 mmol/L arginine) to stimulate bone marrow derived macrophages (BMDMs). In comparison to HEK293T cells, only leucine induced the phosphorylation of S6, 4-EBP1 and p70S6K in macrophages but to a lesser extent (Fig. 1A). Neither glutamine nor arginine induced any detectable mTORC1 activation, indicating amino acid-induced mTORC1 activation is cell-type specific (Fig. 1B and 1C).

**Figure 1:**
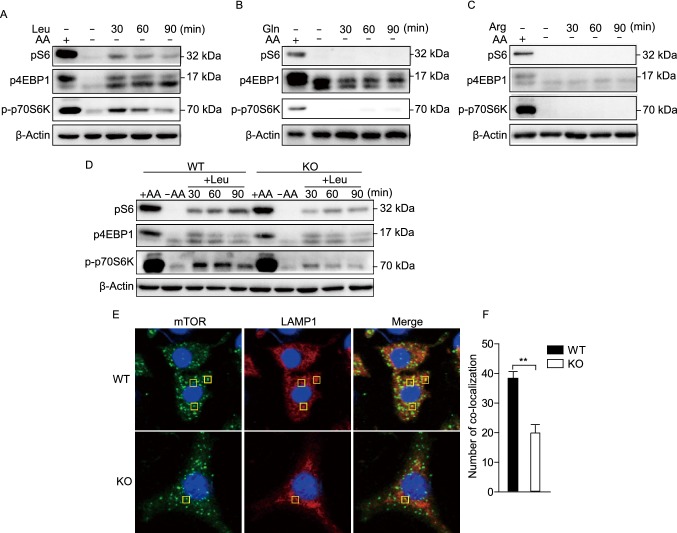
**Leucine, but not glutamine and arginine, induces mTORC1 activation in macrophages**. BMDMs were starved with DMEM medium containing no amino acids and FBS for 2 h, followed with stimulation with medium containing AAs or 4 mmol/L leucine (A), 20 mmol/L glutamine (B), and 2 mmol/L arginine (C) for the indicated times. Phosphorylation S6, 4E-BP1, p70S6K, and β-actin were determined by immunoblotting analysis. (D) Wild type and *Atp6v0d2*^−/−^ BMDMs were starved with DMEM medium containing no amino acids and FBS for 2 h, followed with stimulation with DMEM containing 4 mmol/L leucine for the indicated times. Phosphorylation S6, 4EBP1, p70S6K, and β-actin were determined by immunoblotting. (E) Wild type and *Atp6v0d2*^−/−^ BMDMs were starved with DMEM medium containing no amino acids and FBS for 2 h, followed with stimulation with medium containing 4 mmol/L leucine for 30 min. Lysosomes were visualized by immunostaining with antibodies to LAMP-1, nuclei were visualized by DAPI, and mTOR was visualized by immunostaining with antibodies to mTOR. Quadrilaterals indicate co-localization of LAMP1 and mTOR. (F) The numbers of co-localizations of LAMP1 with mTOR in 5 fields of view were quantified. Data shown are representative of three independent experiments. Bars = mean ± SEM, **P < 0.01 (Students’s paired *t*-test)

To investigate the possible function of ATP6V0d2 in amino acid-induced mTORC1 activation, we compared leucine-induced mTORC1 activation in WT and *Atp6v0d2*^−/−^ BMDMs. Deletion of ATP6V0d2 resulted in impaired phosphorylation of S6, 4-EBP1, and p70S6K upon leucine treatment in macrophages, despite comparable mTORC1 activation upon complete amino acids stimulation (Fig. 1D), indicating that ATP6V0d2-independent activation of mTORC1 induced by other amino acids may exist. Conversely, overexpression of ATP6V0d2 enhanced leucine-induced mTORC1 activation measured by S6 phosphorylation (Fig. S1D). Immunofluorescence staining showed that absence of ATP6V0d2 reduced the co-localizations of mTOR and LAMP1 (Fig. 1E and 1F), a lysosome marker, indicating that ATP6V0d2 may promote the recruitment of mTOR to the lysosome membrane.

Given the role of mTORC1 in macrophage polarization (Byles et al., 2013), next we compared the macrophage polarization under M1 and M2 conditions between WT and *Atp6v0d2*^−/−^ BMDMs. The expression of M1-associated genes *Tnfa* and *iNOS* was reduced in LPS and IFN-γ polarized *Atp6v0d2*^−/−^ BMDMs, compared to WT counterparts (Fig. S2A and S2B). This was associated with impaired phosphorylation of S6, 4-EBP1 and p70S6K in *Atp6v0d2*^−/−^ macrophages upon LPS and IFN-γ stimulation (Fig. S2C). In contrast, the expression of M2-associated genes *Arginase-1*, *Fizz-1* and *Ym-1* was enhanced in IL-4-polarizaed *Atp6v0d2*^−/−^ BMDMs, compared to WT cells (Fig. S2D–F). Furthermore, deletion of ATP6V0d2 significantly suppressed the macrophage polarization into F4/80^+^CD11c^+^ M1 phenotype (Fig. S3A and S3B); in contrast, the polarization into F4/80^+^CD206^+^ M2 phenotype was enhanced (Fig. S3C and S3D). The expression of another M2 marker CD301 upon IL-4 stimulation was also enhanced in the absence of ATP6V0d2 (Fig. S3E and S3F). These data indicate that ATP6V0d2 promotes M1 polarization but suppresses M2 polarization of macrophages *in vitro*.

To test if ATP6V0d2 plays a role in the regulation of mTORC1 activation and macrophage polarization *in vivo*, wild-type and *Atp6v0d2*^−/−^ mice were fasted for 16 h and administrated with leucine for 1 h for detection of mTORC1 activation or twice a day for 48 h for measuring the macrophage polarization. There was no difference in the phosphorylation of S6 in splenic F4/80^+^ macrophages between wild-type and *Atp6v0d2*^−/−^ mice upon starvation (Fig. 2A). However, after administration of leucine, the phosphorylation of S6 in macrophages from *Atp6v0d2*^−/−^ mice was significantly reduced compared with macrophages from control animals (Fig. 2B and 2C). Consistent with the *in vitro* data, deletion of ATP6V0d2 significantly reduced splenic F4/80^+^CD11c^+^ M1 polarization but enhanced F4/80^+^CD206^+^ M2 polarization upon leucine gavage (Fig. 2D–G). The expression levels of M1-associated genes *Tnfa* and *iNOS* were reduced in the F4/80^+^ splenic macrophages from *Atp6v0d2*^−/−^ mice, compared to WT counterparts (Fig. 2H and 2I). Conversely, the expression levels of M2-associated genes *Arginase-1*, *Fizz-1* and *Ym-1* were enhanced, in the F4/80^+^ splenic macrophages from *Atp6v0d2*^−/−^ mice (Fig. 2J–L). These data suggest that ATP6V0d2 regulates leucine-induced mTORC1 activation and macrophage polarization *in vivo*.

**Figure 2:**
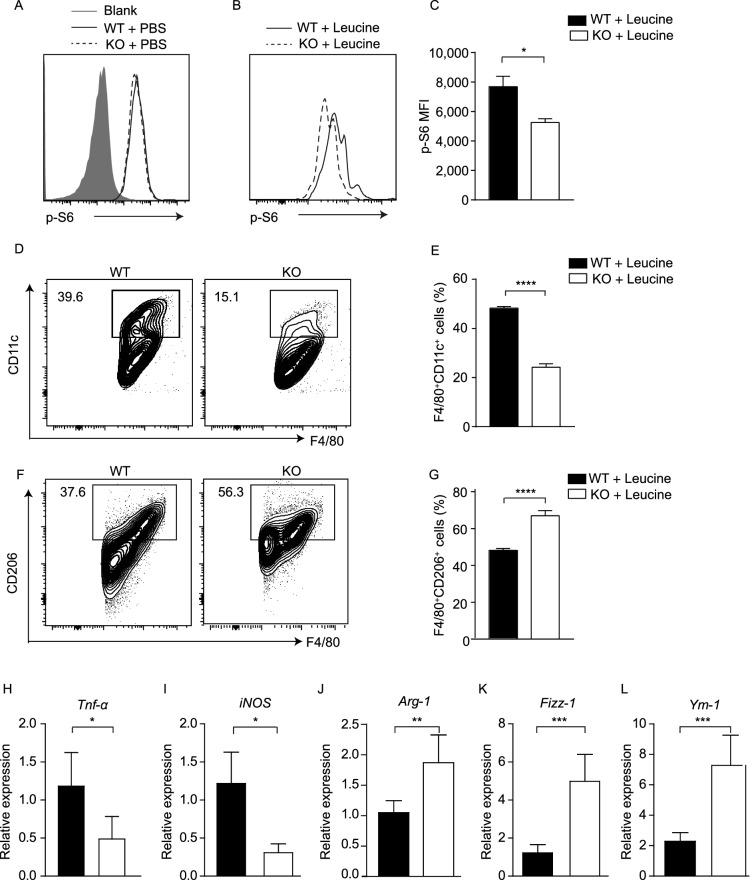
**ATP6V0d2 regulates leucine-induced mTORC1 activation and macrophage polarization** ***in vivo***. Wild type and *Atp6v0d2*^−/−^ mice (*n* = 5) were starved for 16 h, and then gavaged with 200 μL PBS (A) or 200 μL leucine (54.0 g/L in PBS) (B and C). After 1 h, the S6 phosphorylation of splenic macrophages was determined by flow cytometry. Wild type and *Atp6v0d2*^−/−^ mice were starved for 16 h, and then gavaged with 200 μL leucine (54.0 g/L) twice a day for 48 h. After 6 h the last gavage, the expressions of F4/80 and CD11c (D and E) or CD206 (F and G) of splenocytes were determined by flow cytometry. The expressions of *Tnf-a* (H), *iNOS* (I)*, Arginase-1* (*Arg-1*) (J), *Fizz-1* (K) and *Ym-1* (L) of F4/80^+^ macrophages were determined by RT-PCR. Data shown are representative of three independent experiments for leucine-induced mTORC1 activation. Bars = mean ± SEM, *P < 0.05, **P < 0.01, ***P < 0.005, ****P < 0.0001 (Students’s paired *t*-test)

Amino acids engage overlapping and specified components for mTORC1 activation (Efeyan et al., 2015). It is not clear why only leucine induces mTORC1 activation in macrophages. Macrophages are critical sentinel for immune responses and tissue homeostasis (Ginhoux and Guilliams, 2016). One possibility is that macrophages lack proteins that are required for sensing arginine and glutamine. We speculate that unresponsiveness to arginine and glutamine may help prevent unwanted activation of macrophages. The current study did not completely elucidate the mechanism by which ATP6V0d2 regulates leucine-induced mTORC1 and this question warrants further investigation. Interestingly, ATP6V0d2 also promotes mTORC1 activation under M1 macrophage polarization. We previously showed that ATP6V0d2 does not regulate lysosome acidification, which is consistent with other studies (Lee et al., 2006). It is highly likely that ATP6V0d2 serves as an adaptor protein that facilitates the interaction between mTORC1 and other critical protein complexes i.e., Rag GTPases or Ragulator. Our previous study implied that macrophage specific ATP6V0d2 might exist in other protein complexes independent of V-ATPase (Xia et al., 2019). *Atp6v0d2*-deficient BMDMs have reduced M1 differentiation but enhanced M2 differentiation, which is consistent with enhanced presence of M2 macrophages within tumors in *Atp6v0d2*-deficient mice in xenograft tumor model (Liu et al., 2019). In line with our results, constitutive activation of mTORC1 in the *Tsc1*^−/−^ BMDMs inhibits M2 polarization but promotes M1 polarization; mice with myeloid specific *Tsc1* deficiency spontaneously develop inflammatory disorders (Zhu et al., 2014). These data highlight that the amount of mTORC1 activation might be a critical parameter in the determination of differentiation and functions of immune cells, which is consistent with previous studies (Hukelmann et al., 2016).

In summary, here we identified that ATP6V0d2 mediates leucine-induced mTORC1 activation in macrophages, which further regulates macrophage differentiation. These data demonstrate a cell-specific role of V-ATPase subunit in mediating amino-acid-induced mTORC1 activation.

## Electronic supplementary material

Below is the link to the electronic supplementary material.

Supplementary material 1 (PDF 571 kb)
