# Supplementary material for: ATP6V0d2 mediates leucine-induced mTORC1 activation and polarization of macrophages
Source: Protein Cell. 2019 May 27;10(8):615–9. doi: 10.1007/s13238-019-0636-x (PMC6626592; doi:10.1007/s13238-019-0636-x)
Supplement: Supplementary file 1 — Supplementary material 1 (PDF 571 kb) [file 13238_2019_636_MOESM1_ESM.pdf]

ATP6V0d2 mediates leucine-induced mTORC1 activation in macrophages

Pingfei Li[1,2], Xiaofei Deng[1], Jing Luo[1,3], Yufei Chen[1], Guoyu Bi[1], Feili Gong[1], Zhengping Wei[1], Na Liu[1], Huabin Li[4], Arian Laurence[5], Xiang-Ping Yang[1*]

Supplementary Materials

Materials and methods

Mice

Atp6v0d2$^{-/-}$ mice were generated using TALEN (transcription activator-like effector nuclease) technology as previously described (Liu et al., 2019). C57BL/6 WT mice were purchased from Huafukang (Beijing). All animal studies were approved by the Animal Experiment Committee of Huazhong University of Science and Technology and in accordance with the guidelines of school of basic medicine, Tongji Medical College.

To measure mTORC1 activation, 8-week wild type or *Atp6v0d2*$^{-/-}$ mice (20 g) were fasted for 16 h, and then gavaged with 200 μl PBS or the equivalent volume of 54.0 g/L leucine. After 1 h, mice were sacrificed and S6 phosphorylation of splenic macrophages was determined by flow cytometry. For the detection of leucine-induced macrophage polarization, 8-week wild type or *Atp6v0d2*$^{-/-}$ mice (20 g) were fasted for 16 h, and then gavaged with 200 μl leucine (54.0 g/L, twice a day) (Anthony et al., 2000) for 48 h. After 6 h the last gavage, mice were sacrificed and polarization of splenic macrophages was determined by flow cytometry or RT-PCR.

Cell culture

Primary bone marrow derived macrophages (BMDMs) were generated by culturing mouse bone marrow cells in the presence of 50 ng/ml M-CSF (Peprotech, Cat. 315-02) conditional medium for 7 days. On day 7, macrophages were washed and stimulated with LPS (100 ng/ml) plus IFN-γ (20 ng/nl) for the indicated times.

Amino acid starvation and stimulation of cells

Amino acid free DMEM was house made following the Invitrogen high glucose DMEM recipe (Cat: 11965-092) with the exception that all amino acids were omitted. Leucine (Sigma, L8912), glutamine (Sigma, G8540) and arginine (Sigma, A8094) were added to amino acid free medium, prepared in-house to the final indicated concentrations. Cells were starved with DMEM medium containing no amino acids and FBS for 2 hours prior to stimulation. Subsequently, cells were stimulated with either all amino acids, or 4 mM leucine, 20 mM glutamine, 2 mM arginine individually for the indicated times. After stimulation, cells were harvested and cell lysates were prepared.

Western blot

Cells were lysed in RIPA buffer (25 mM Tris-HCl, pH 7.6, 150 mM NaCl, 1% NP-40 (Sigma Aldrich, NP40S), 1% sodium deoxycholate (Sigma Aldrich, D6750), 0.1% SDS (Sigma Aldrich, 74255) containing protease inhibitor cocktail (Roche, 11836153001) and phosphatase inhibitor cocktail (Sigma Aldrich, P5726). Protein concentrations were determined using a BCA Protein Assay Kit (Pierce, 23225).

Equal amounts of protein were loaded onto SDS-PAGE gels and transferred onto nitrocellulose membranes. The membranes were then incubated with antibodies against p-S6 (CST, 4858), p-4EBP-1 (CST, 9459), ATP6V0d2 (Sigma, SAB2103221) and β-Actin (CST, 3700), followed by appropriate secondary HRP-conjugated Goat Anti-Rabbit IgG, then developed with ECL (GE healthcare, RPN2232).

Plasmid transfection

Control Vector (pENTER) and pENTER-ATP6V0d2 plasmid (Vigenebio, CH861157) were transfected into HEK293T cells using Lipofectamin 2000 according to the manufacturer's instructions.

Immunofluorescent staining and confocal microscopy

BMDMs from WT and *Atp6v0d2*$^{-/-}$ mice were seeded at 2×10^4 cells per well on glass slides, and rested overnight for proper attachment. The cells then underwent 2 hours of amino acid starvation followed by 30 mins of leucine treatment. After treatment, cells were washed twice with sterile PBS and fixed with 4% paraformaldehyde (PFA), permeabilized with 0.05% Triton X-100 and blocked in 5% BSA. Anti-LAMP1 (BD Bioscience, 555798) and anti-mTOR (CST, 2983T) were incubated overnight at 4°C. Secondary fluorescent antibodies were added for 1 hour and DAPI was used for nuclear counterstaining. Samples were imaged through a laser scanning confocal microscope (Olympus, Japan).

Flow cytometry

BMDMs or splenocytes from WT and *Atp6v0d2*$^{-/-}$ mice were cultured with or without 100 ng/ml LPS (Sigma, E.coli O111:B4) and 20 ng/ml murine IFN-γ (R&D, 485-MI) or 20 ng/ml murine IL-4 (R&D, 404-ML) for 24 hours. After Fc blockade with anti-CD16/CD32 (clone 93), cells were stained with following antibodies: CD11c-PE (clone N418), F4/80-PerCPcy5.5 (clone BM8), CD206-PE/cy7 (clone C068C2) and CD301-APC (clone URA-1) (all from Biolegend). For phosphor flow staining, 2×10^6 splenocytes from WT or *Atp6v0d2*$^{-/-}$ mice were fixed with BD Phosflow™ Fix buffer (BD Bioscience, 557870) for 20 mins, then blockade Fc receptor with anti-CD16/CD32. Cells were stained with F4/80-PerCPcy5.5 and Phospho-S6-PE (CST, 4858). Samples were collected on a BD Verse Flow cytometer, and data were analyzed using FlowJo software.

Isolation macrophages

5×10^7 splenocytes from WT or *Atp6v0d2*$^{-/-}$ mice were incubated with biotin-anti-F4/80 antibody (Biolegend, 123106) for 30 mins, then stained with anti-biotin MACSi Bead (Miltenyi , 130-091-147) for 20 mins. F4/80$^+$ cells were isolated with LS magnetic beads.

Quantitative RT-PCR

Total RNAs were extracted from BMDMs or F4/80$^+$ macrophages with TRIzol reagent (Invitrogen). cDNA was synthesized from 1 μg RNA using the Reverse Transcription Kit (Toyobo, Japan) following the manufacturer's protocol. All quantitative RT-PCR was performed by the SYBR green method on a Bio-Rad CFX

connect. The quantification of the results was performed by the comparative Ct ($2^{-\Delta\Delta Ct}$) method. The Ct value for each sample was normalized by the value for *Actin* gene. Primers sequences for the genes are followed:

Gene	Forward primer 5'— 3'	Reverse primer 5'— 3'
Tnf-α	TACTGAACTTCGGGGTGATTG	CAGCCTTGTCCCTTGAAGAGAA
iNOS	CGAAACGCTTCACTTCCAA	TGAGCCTATATTGCTGTGGCT'
Arg-1	CGTGGGTCCAACACCCTTAT	TGGTCTGCTATTTGCCAGGA
Fizz-1	CAAGACTATGAACAGATGGGC	AGGAGATTGATGGGAGAGGAC
Ym-1	GAAGGAGCCACTGAGGTCTG	CACGGCACCTCCTAAATTGT
Actin	TCTGAATGTATTTGGGTGACTC	AACGCAGCTCAGTAACAGTCC

Statistical Analysis

All data are shown as means ± s.d. or means ± s.e.m. as indicated. Statistical analysis was performed using a two-tailed Paired Student's *t*-test. For all the tests, *p*-values lower than 0.05 were considered significant.

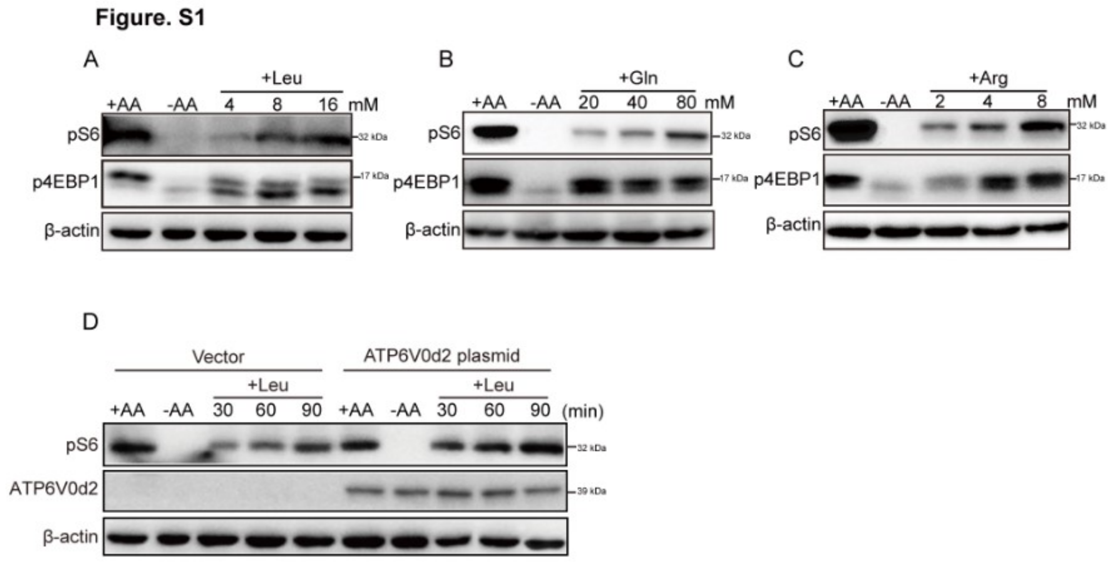

Figure S1. ATP6V0d2 promotes mTORC1 activation in HEK293T cells. HEK293T cells were starved with DMEM medium containing no amino acids and FBS for 2 hours, followed with stimulation with medium containing AAs or indicated concentrations of leucine (Leu) (A), glutamine (Gln) (B), and arginine (Arg) (C) for 30 mins. Phosphorylation S6, 4E-BP1 and β-actin were determined by immunoblotting analysis. (D) HEK293T cells were transfected with vector or pENTER-ATP6V0d2 plasmids. Cells were starved with DMEM containing no amino acids and FBS for 2 hours, followed with stimulation with medium containing 4 mM leucine for the indicated times. Phosphorylation S6, ATP6V0d2 and β-actin were determined by immunoblotting. Data shown are representative of three independent experiments.

Figure. S2

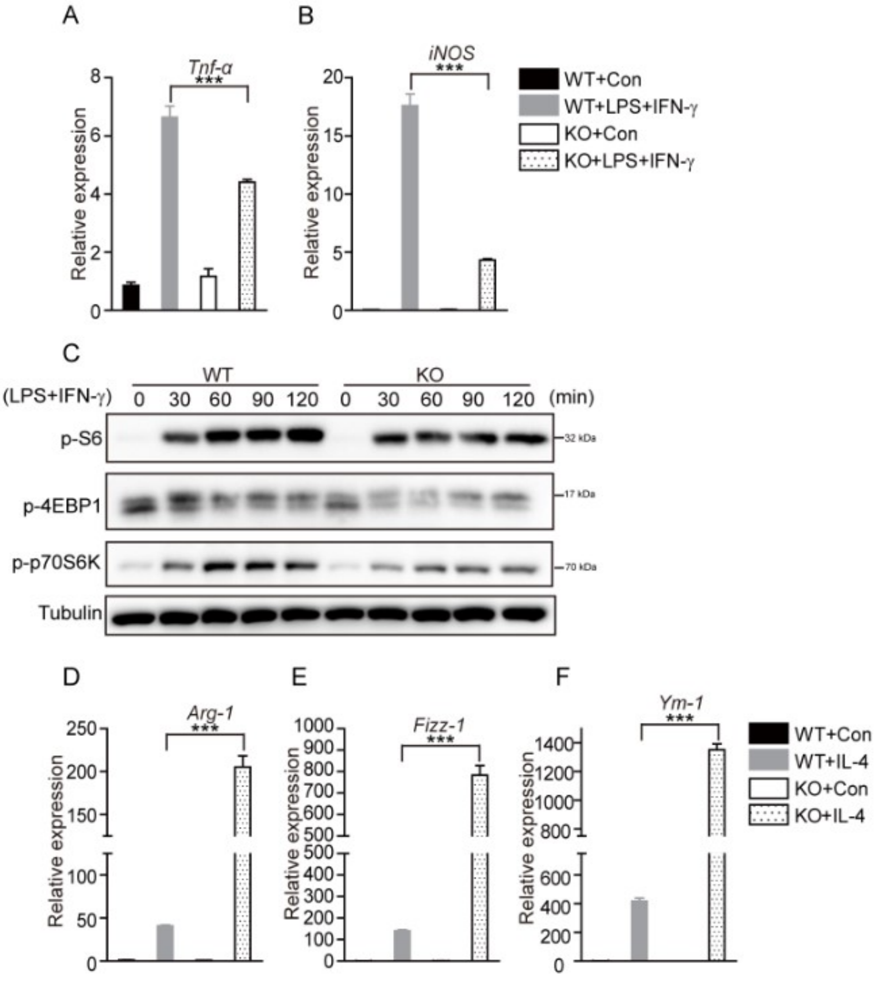

Figure S2. Deletion of ATP6V0d2 leads to enhanced M2 polarization. Wild type and *Atp6v0d2*$^{-/-}$ BMDMs were polarized under M1 conditions (100 ng/ml LPS and 20 ng/ml IFN-γ) (A-C). After 12 hours, the expressions of *Tnf-α* (A) and *iNOS* (B) were analyzed by RT-PCR. After stimulation for the indicated times, the phosphorylation S6, 4E-BP1, p70S6K and β-actin were determined by immunoblotting analysis (C). Wild type and *Atp6v0d2*$^{-/-}$ BMDMs were polarized under M2 conditions (20 ng/ml IL-4) for 12 hours (D-F). The expressions of *Arginase-1 (Arg-1)* (D), *Fizz-1* (E) and *Ym-1* (F) were determined by RT-PCR. Data shown are representative of three independent experiments. (Student's paired *t*-test).

Figure. S3

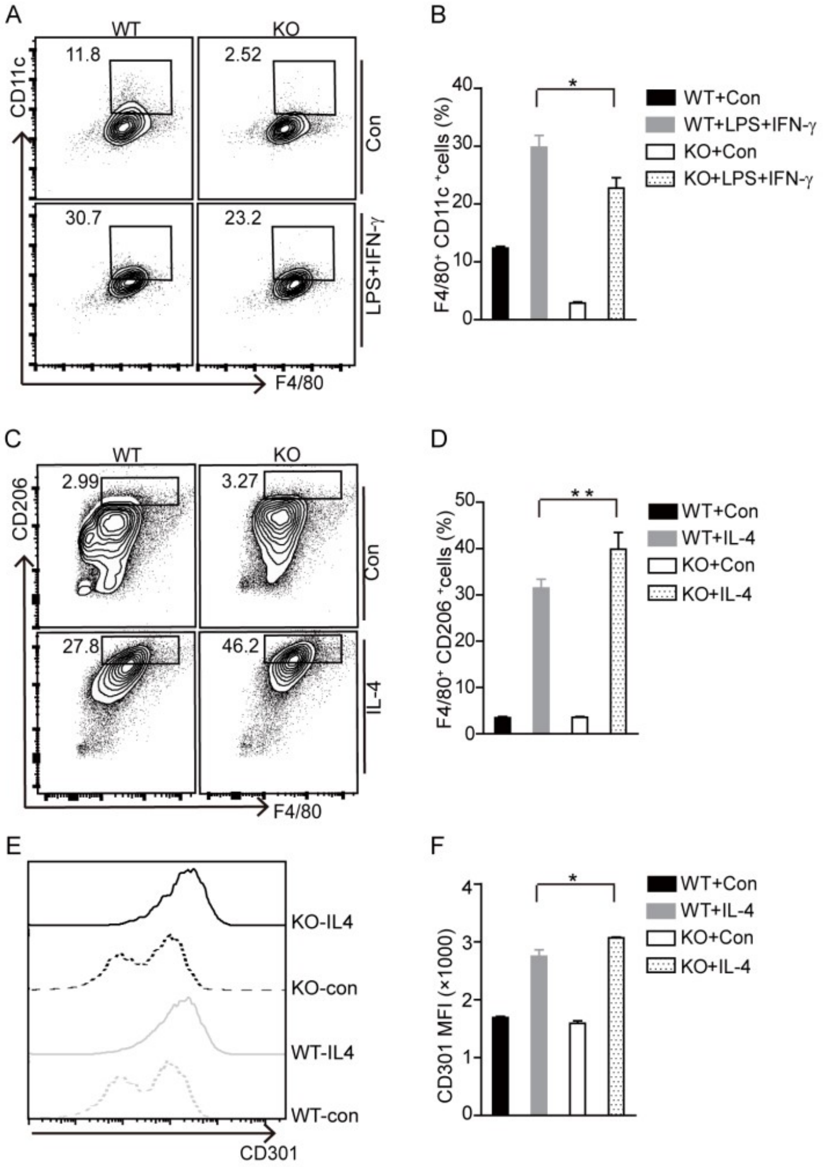

Figure S3. Deletion of ATP6V0d2 leads to enhanced M2 polarization. Wild type and *Atp6v0d2*$^{-/-}$ BMDMs were polarized under M1 conditions (100 ng/ml LPS and 20 ng/ml IFN-γ) (A, B) or M2 conditions (20 ng/ml IL-4) (C-F). After 24 hours, the expression of F4/80 and CD11c, CD206 or CD301 was determined by flow cytometry. Data shown are representative of three independent experiments. (Student's paired *t*-test).

REFERENCES

Anthony, J.C., Yoshizawa, F., Anthony, T.G., Vary, T.C., Jefferson, L.S., and Kimball, S.R. (2000). Leucine stimulates translation initiation in skeletal muscle of postabsorptive rats via a rapamycin-sensitive pathway. J Nutr 130, 2413-2419.

Liu, N., Luo, J., Kuang, D., Xu, S., Duan, Y., Xia, Y., Wei, Z., Xie, X., Yin, B., Chen, F., *et al.* (2019). Lactate inhibits ATP6V0d2 expression in tumor-associated macrophages to promote HIF-2alpha-mediated tumor progression. J Clin Invest 129, 631-646.
